# Effect of repeated autoclaving on implant abutments from genesis and bredent dental implant systems

**DOI:** 10.6026/973206300191419

**Published:** 2023-12-31

**Authors:** Patel Ila Rajendra, Ramchandra Kore Abhijeet, Sanyal Pronob Kumar

**Affiliations:** 1 Department of Prosthodontics and Crown and Bridge, School of Dental Sciences, Krishna Institute of Medical Sciences (KIMS-DU), Karad, Maharashtra, India

**Keywords:** Surface roughness, titanium abutments, sterilization

## Abstract

The effect of repeated autoclaving on the implant-abutment connection of titanium abutments from Genesis and Bredent dental implant systems is of interest to dentists. 40 screw-retained titanium implant abutments from Genesis and Bredent were divided into four groups of ten. Each implant was secured with an abutment using screws. Abutments were prepared for the first 30-minute autoclave cycle at 121°C. After the first autoclave cycle, the abutments were fitted onto their implant systems and examined under a scanning electron microscope (SEM). Intra-group comparison between marginal gaps of Genesis and Bredent groups at 1st autoclave and 2nd autoclave observed statistically significant differences respectively (p<0.05). Genesis group showed highest mean values for buccal and mesial sides (2.7) and lingual and distal sides (2.8) with statistically significant differences. Marginal gap and surface roughness increased with autoclaving for both Genesis and Bredent group of implant abutment systems.

## Background:

Implant dentistry is one of the most critical revolutions which occurred in the field of dentistry [1, 2]. When compared to the wide number of treatments that are available in the field of dentistry, implant therapy stands out due to the positive outcomes it produces. Titanium implants at the superior most position of this triad. The two-piece implant system, which consists of the implant body and the abutment, is the type of dental implant system that is utilized the most frequently overall. The component of a dental implant that is instrumental in providing support and/or retaining prosthesis is referred to as an abutment [3,4]. Despite the fact that there is a wide range of materials that can be employed for implant abutment, titanium abutments are the ones that are utilized the most frequently. One of the most significant synaptic connections between the implant body and the abutment that is utilized is the connection that is formed between the implant abutments [5]. A significant milestone for the colonization of microorganisms can also be considered to be the link that exists between the implant and the abutment joint. It is typically characterized by the presence of a microgap, which is characterized by differences in both the vertical and horizontal dimensions between the surface of the abutment and the surface of the implant [6,7]. Even though it is reported that the success rate of any osseo-integrated implant is approximately 90 percent, there is still the possibility that the implant-abutment system could experience certain mechanical issues, which could result in the implant failing. Mechanical problems at the implant-abutment connection can even lead to microbial proliferation into these areas and cause inflammation around the implant soft tissues leading to peri-implantaitis [8]. In order to ensure the stability of the connection between the implant body and the abutment, it is essential that the abutment and implant body have a high degree of precision. Sterilization of abutment following lab or clinical use is a common practice in a clinical setting. It is the process of eliminating microbial viability. Biological load reduces after sterilization, but sometimes these procedures might lessen the material's ability to withstand mechanical stress. Repeated adjustments and the use of abutments require repeated sterilization. During the sterilization of abutments, multiple autoclave cycles might cause changes in the surface and other mechanical properties [9]. Sterilization has proven to reduce the biological microbial load from the abutment and implant surface, but the mechanical changes associated with these procedures are yet to be studied. Therefore, it is of interest to report the effect on these titanium abutments due to repeated sterilization cycles

## Materials and Method:

The Genesis and Bredent implant-abutment systems were the subjects of an in-vitro investigation. There was a successful acquisition of both scientific and ethical approval from the institution. Forty screw-retained titanium implant abutments were included in the study. Twenty of these abutments were identified as belonging to the Genesis group, while the remaining twenty were identified as belonging to the Bredent group. A total of ten abutments were distributed across the four distinct groups that were segmented. In order to attach these abutments to their corresponding implants, screws were used. Within the scope of this study, four implants were included, each of which had the identical dimensions, specifically 3.5 mm by 10 mm, for each of the four groups. Two implants were used from the Genesis implant system and two for the Bredent implant system, respectively, for connecting their respective abutments. Genesis Aktiv Implants were used with Genesis Aktiv Straight Universal abutments(2mm). Bredent SKY Implants with Bredent SKY exso Straight Universal abutments (2mm) were used in this study.

## Study procedure:

A three-dimensional model of the premolar and molar mandibular arch was produced in Pixologic Zbrush 2.0 utilising a CBCT report of the edentulous arch. Its virtual design and curation created a conventional mandibular arch. The 1st molar region was sectioned into 1cm x 1cm model blocks prepared for individual implants. This model's requirements were used to create and manufacture the CAD model. Four blocks were designed to receive four implants from four groups. The mandibular arch form components were created and 3D printed.

SLA technology was used to produce a typical study model with 100% infill density from this stereolithography (.stl) CAD design using 3D Printer Form 2 Basic (Formlabs, USA). Stereolithography, or "SLA," is an additive manufacturing technology that typically uses an ultraviolet (UV) laser on photopolymer resin. Sectional mandibular arch 3D models were printed. Three-dimensional printing produced four blocks, two per group. Implants were then drilled on these 3D sectional arch model of mandibular arch manufactured using CAD-CAM and 3D printing.

Perpendiculars from buccal, lingual, and mesial, distal sides identified the implant locations to find the resin model's centre. This provided the exact center and even placement of implant in each of the resin models. Genesis Aktiv implant drilling kit was used for drilling Genesis Aktiv implants. Initially, a pilot drill (2.3/2.0 mm, Short, PD2.3S) was utilised up to 8mm of osteotomy height, followed by successive surgical drills (2.8/2.3mm, Short SD 2.8S) and a surgical drill (3.4/2.8mm, Short, SD 3.4S) to reach 10mm. Later implant was placed in the prepared osteotomy site using an implant driver (2.4 mm, Short, IDS) manually. Similarly, Utilising sequential drilling, Bredent SKY implants were inserted into the resin model during the process. A pilot drill (SKYDP08) was used to start the osteotomy site preparation followed by a surgical drill (SKYD3435). An implant driver (SKY-STK6) was used to place the implant in the prepared site manually. With the use of a torque ratchet, implants for both of the systems were inserted into the model, and the final torque was measured at 35Ncm.

After the implants were screwed onto their respective models, four areas were designated on the mounted implant model. These areas were buccal, mesial, palatal, and distal. Boundaries were marked along the model using four dots or notched onto the surface of the model to facilitate scanning [10]. In order to create these notches, a fine diamond round bur and turbine were utilised while a stereomicroscope was being utilised. The use of permanent colour markers allowed for the color-coding of each of these four spots. Buccal is red, mesial is green, lingual is blue, and distal is yellow so that the side can be recognised more easily and scanning may be performed sooner [10].

## Autoclave cycles:

The third and fourth study groups were defined by the presence of ten Genesis Aktiv Straight Universal abutments with a dimension of 2 millimetres and ten Bredent SKY exso Straight Universal abutments with the same dimension. Wrapping and packing these abutments with Oro Sterilisation Reels - 100MM heat sealing flat reel was done in order to get them ready for the first cycle of an autoclave. To guarantee that the sterilisation procedure was carried out correctly, the autoclave kept the temperature at 121 degrees Celsius for at least thirty minutes while applying saturated steam at a pressure of at least fifteen pounds per square inch. Following the completion of the first cycle of the autoclave, the abutments belonging to the third and fourth groups were screwed onto their respective implant systems and examined using a scanning electron microscope. A second cycle of autoclaving was performed on the identical sets of implants that were used in the third and fourth groups. After that, the implants were examined once again using a scanning electron microscope to look for any modifications that may have occurred. The abutment from each group was screwed onto the dental implant using a torque of 35 Ncm for the Genesis group and 35 Ncm for Bredent Group, following the manufacturers' guidelines.

## Digital Scanning:

Group 1 and group 2 were the control groups used in this study. A scanning electron microscope was used to scan each of these specimens. An examination was carried out on the abutment's surface modifications as well as the vertical gap. Using a K650 sputter coater from Quorum Technologies, each specimen was gold-sputtered, and a scanning electron microscope was utilised to get an image of the implant-abutment gap at the marginal contact [11]. The implant abutment connection was maintained in a parallel position to the detector of this SEM equipment. This was done to guarantee that the sample was positioned correctly and that the vertical gap analysis was performed correctly. Additionally, the lingual and distal aspects were scanned periodically. An image of the marginal fit was obtained for every facet of each specimen by obtaining a scan in a perpendicular direction using a magnification of 1000 times at a predetermined angle. Image analysis software (Vega3 TESCAN) was utilised to perform three vertical marginal gap assessments for each and every scan. The mean value for each side was determined by calculating the average of these three measurements. Therefore, two measurements were taken for each specimen, and each side of each specimen was measured twice. The surface detection programme (Vega3 TESCAN) was utilised for the purpose of surface analysis in order to validate the surface roughness.

Group 3rd and group 4th were autoclaved for 1st time with the standard protocol in consideration in the Tabletop front-loading autoclave unit. Each of these specimens was scanned using a SEM with a similar procedure used to scan group 1st and 2nd. Group 3rd and group 4th were again autoclaved for 2nd time with the standard protocol in the tabletop front-loading autoclave unit. Each of these specimens was scanned again with SEM with a similar procedure used to scan group 1st and 2nd.

## Digital Analysis:

## Assembly:

After all of the samples had been scanned, they were gathered together and compiled into a jpg file. After that, each and every photograph was examined to determine whether or not there were any mistakes. After each of the photographs had been examined, they were further subdivided into the portions that were most pertinent to them.

## Sectioning:

After assembling the samples, they were sectioned into respective groups. Four different groups compromising of scanned images for group 1 (control) included pre-sterilized Genesis titanium abutments. group 2 (control) included scanned images of pre-sterilized Bredent titanium abutments. Group 3 included scanned images of autoclaved Genesis titanium abutments. Group 4 included scanned images of autoclaved Bredent titanium abutments. Sectioning of this sample data into respective groups provided aid in measuring the samples correctly with ease.

## Measurements:

For the purpose of measuring the vertical marginal disagreement and surface roughness, each of the four groups was evaluated independently. For the purpose of taking vertical marginal measurements, a numerical measuring tool was utilised within the software scale 2.3, Quartz PCI, version 5.5, which was developed by Quartz Imaging Corporation. The vertical measurement was carried out at three distinct locations, and the final vertical marginal gap was determined by taking the average of these three readings. All of the surface roughness measurements were carried out using the surface detection software (Vega3 TESCAN). Digital analysis was performed on the surface of the particular abutment by utilising the software that was included inside the device, which was based on pixel capture.

## Statistical Analysis:

The data was entered and analyzed using Statistical Package for Social Sciences (SPSS) for Windows 26.0 (SPSS, Inc. Chicago, Illinois). Confidence intervals were set at 95%, and a p-value ≤ of 0.05 was considered as statistically significant. Unpaired t test was applied to compare Genesis and Bredent groups at 1st autoclave, 2nd autoclave and control between both groups. Repeated measures ANOVA was used to check the significance of difference in Genesis and Bredent at control, 1st autoclave and 2nd autoclave.

## Results:

**Figure 1:**
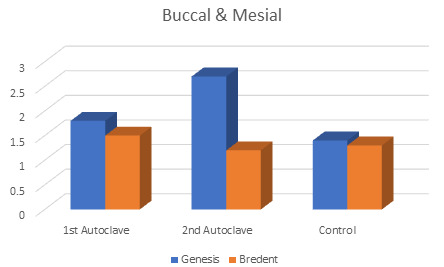
Marginal gaps in both the groups: Buccal and Mesial

**Figure 2:**
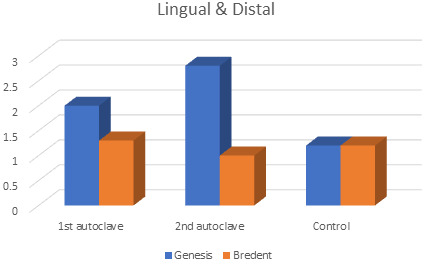
Marginal gaps in both the groups: Lingual and Distal

**Figure 3:**
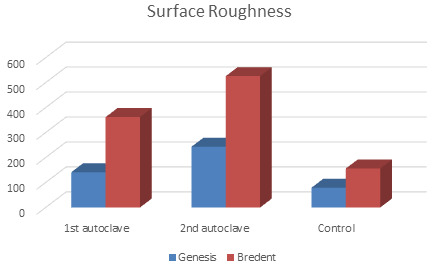
Surface Roughness in both the groups

Intragroup comparison between marginal gaps on buccal and mesial sides of Genesis and Bredent groups at 1st autoclave and 2nd autoclave showed statistically significant difference respectively. (p<0.05) Statistically significant difference (p<0.05) was seen in Genesis group with the highest mean values (2.8) at the 2nd autoclave. (Figure 1) Intragroup comparison between marginal gaps on lingual and distal sides of Genesis and Bredent groups at 1st autoclave and 2nd autoclave showed statistically significant difference respectively. (p<0.05) Statistically significant difference (p<0.05) was seen in Genesis with the highest mean values (2.8) at the 2nd autoclave. (Figure 2) There was no significant difference seen with Bredent group. Surface roughness for Genesis (243.7) and Bredent groups (528) was highest at the 2nd autoclave. Statistically significant difference (p<0.05) was seen with intergroup and intragroup comparison for surface roughness in both the groups. (Figure 3)

## Discussion:

There is a correlation between the characteristics of the surface and the quality of dental implants. A significant role was played by the biocompatibility of the materials and the roughness of the surface in order to achieve a successful interaction between the tissue and the osseointegration process [12]. There is a wide variety of sterilisation techniques available, each of which operates in a distinct way. The physical method of sterilisation known as autoclaving (AC) is considered to be the gold standard. This process exposes living organisms to circumstances of temperature, pressure, and duration that are not sustainable. There is a possibility that the effectiveness of the autoclave will change depending on the density, volume, and size of the material of interest. There is also the possibility of employing chemical treatments, such as oxygen plasma (OP). In this technique, an ionised gas is used to bombard the surface of the substratum, which in turn encourages the production of free radicals in an atmosphere of vacuum. When active species, such as polar groups, are present, they degrade and remove the surface layer [13]. The type of gas, the purity of the gas that is supplied, the pressure that is applied to the gas, and the position of the sample can all have an effect on the thickness of the layer that is removed as well as the new surface attributes [14]. Also, if the interface of the implant with its respective prosthetic connection is not precise, it can result in a change in the microbiological parameters, as bacterial growth could subsequently compromise the periodontal tissues that are adjacent to the implant. The presence of germs that contaminate the interior section of osseo-integrated implants has the potential to cause contamination of the implant or abutment during the two stages of surgical procedures [15].

One of the most critical factors that determine whether or not implant therapy is successful is the surface roughness of the implant abutment component. One of the most crucial characteristics of bacterial adhesion and colonisation is the roughness of those hard surfaces that are found inside the mouth. An accumulation of subgingival plaque can be up to 25 times more on rough surfaces than it is on smooth locations [16]. For abutments, it is commonly preferable to have "smooth" surfaces with roughness values that are smaller than the "critical threshold" of Sa = 0.2 µm (arithmetical mean height). This is due to the fact that it has been established that roughened surfaces lead to an increase in plaque formation in vivo [17]. As bacterial adhesion to intra‐oral, hard surfaces is firmly influenced by the surface roughness of these structures, the effect of repeated sterilization is to be studied with respect to the same. For reusing implant components, primarily with regard to the capability of providing a sterile component, it offers an economic advantage to either the patient or the physician. It is unknown how frequently a reduced fee is distributed, despite the fact that a large number of professionals believe that this practice is carried out for the benefit of the patients. As for the number of implant surgeons that recycle old healed abutments from one patient to the next, it is unknown. However, unless these materials can be cleaned and sterilised in an effective manner, this practice should be evaluated in light of the findings from this study for the following reasons: Firstly, soft tissue integration is influenced by the materials characteristics. In vitro, animal and human studies have all demonstrated titanium and titanium alloy with their biocompatible oxide layer to have the appropriate chemical composition allowing both epithelial cells and connective tissue fibroblasts to adhere, spread, and proliferate. Secondly, it has been observed that surface-free energy is high when the surface is clean, and it is in the opposite direction when the surface is contaminated. When it comes to cell attachment and spreading, the wettability of the surface is considered to be greater when the surface free energy is higher. Surface texture is another factor that might have a significant impact. It has been established that epithelial and human gingival fibroblasts connect and disseminate more quickly on polished surfaces and that cells are sensitive to features as small as (0.2µm) [18] Data shows that both types of the autoclaving process exhibited considerable marginal gaps in both types of implants. The precision of the space in the interosseous implant at the level of the bone crest is associated with a reduction in the formation of inflammatory peri-implant cells and little bone loss, as stated by Broggini *et al.* [19]. The precise assembly of implant components and the precise fit of the prosthesis to the implant are both necessary for long-term life of dental implants and maintenance of bone that supports them. A study that was quite similar to the present study was carried out by Jain and colleagues, who concluded that reusing IHAs numerous times might not be a sensible approach. This is because the microbial colonization and surface alterations that occurs as a result of using this component multiple times might have an impact on the effectiveness of IHAs in soft tissue healing [20]. During the course of the current investigation, the micro-gap was altered by the autoclaving procedure. This change may have been the result of thermal deformation, which might be investigated further.

Jung Hwa Park and colleagues demonstrated that none of the sterilisation techniques had any effect on the surface roughness of the titanium implant. The surface roughness of the SLA (sand blasted or acid etched) titanium implant was affected by AC, OP, and UV sterilization [21]. Another study, Kato *et al.* demonstrated that there were significant differences in the distance and angle when comparing Scanbody connected to the implant before and after the autoclave treatment. However, repeated connections with or without autoclave treatment did not have a significant impact on the surface texture values that were measured [22]. Behr M *et al.* conducted a study to demonstrate the impact that sterilization and ultrasonic cleaning have on the resin cement interface of customized dental implant abutments. They showed that cleaning protocols for customized dental implant abutments that involve the use of ultrasonic as well as autoclave procedures have an effect on the properties of resin cements. Both bonding strength and surface roughness were unaffected [23]. According to Canullo *et al.*, it is important to highlight that the physical process of sterilizing, which involves autoclaving and employing a combination of appropriate heat and pressure, is capable of eliminating all viable forms of microbiota. However, it is not able to successfully remove particle debris from CAD-CAM abutments [24]. In 2022, Lang and colleagues conducted a study in which they evaluated the bond strength between zirconia frameworks and titanium bases by employing a variety of composite resin luting agents. They did this with and without the use of thermo-cycling and autoclaving. The results of the study revealed that autoclaving did not have a significant impact on the bond strength values, and they were found to be acceptable [25].

Data showed significant differences in the marginal gap in both autoclaves other studies reported no differences in the mechanical properties of components sterilization in an autoclave. While sterilisation does have an effect on the mechanical properties of the implant surface, titanium implants are particularly susceptible to this effect. There are studies that have shown that the sterilisation process in the autoclave is responsible for the alteration in the mechanical characteristics that leads to plastic deformation in steel materials when they are subjected to high levels of stress [26,27,28]. Mathew *et al.* examined the mechanical properties of titanium and stainless steel mini-plates before and after repeated autoclaving cycles and found that the values were not constant. Although titanium was tougher, stainless steel had superior tensile and flexural strength. This showed that repeated autoclaving cycles alter mechanical qualities regardless of mini-plate type [28]. This may be due to the titanium oxide dissolution rate is lower than other comparable metals. In stainless steel, corrosion and temperature precipitate carbonates in its microstructure, causing structural weakness, unlike titanium oxide, which is passive. Pure titanium that is sold commercially has a number of mechanical features, including ductility and mechanical properties that are inferior to those of alloys. This metal exhibits a hexagonal structure when it is at room temperature, which is referred to as the alpha phase. These characteristics are related to temperature. At 882 degrees Celsius, the metal exhibits a beta phase, which is a cubic structure with a centred body. This is the first time that the metal undergoes a structural transformation. The titanium is hard and fragile in this last phase, but in the alpha phase, it is ductile and resistant to damage. When compared to the temperatures that are used in the sterilisation process, the temperatures at which titanium undergoes mechanical change are relatively high, at approximately 882 degrees Celsius. In addition to the passage of time and the temperature, there is yet another aspect of the sterilisation process that must be taken into consideration that is humidity. The layer of titanium oxide can become contaminated with ions such as F, Fe, Mg, Si, Cl, N, H, and O as a result of this later process [28].

An implant-abutment assembly will result in micro-gaps and marginal discrepancies. Furthermore, because implant shoulder is situated at level of the alveolar bone crest, the contact between bone and implant is exposed to possible microbial colonisation and is endangered by this possibility. In this regard, it has been demonstrated that the titanium abutment of segmented implant systems might result in the colonisation of microorganisms on the interior of dental implants as a consequence of microbial penetration. Both marginal gap values and surface roughness should be monitoring factors in the eventful success of implant treatment [29].

Heat deformation did cause statistically significant difference in the marginal gap but it is not clinically significant. Increased surface roughness leads to better retention of the crown [30]. So, regular sterilization of abutment can be easily carried out and should be practiced more. The intraoral influence of changes in the marginal gap valves at IAI should be studied further. In vivo analysis with respect to bacterial colonization on the surface of abutment due to changes in the surface roughness was not evaluated. Also, increased numbers of repeated sterilization cycles might influence the marginal gap values and surface roughness of abutment furthermore, which should be studied further.

## Conclusion:

Sterilisation treatment has a particular effect on the marginal and surface properties of implant abutments, which is necessary since implant abutments are required to be modified and used repeatedly. Both the Genesis and Bredent group of implant abutment systems experienced an increase in surface roughness and marginal gap width after being subjected to autoclaving. It has been shown that the surface texture of titanium implants, namely the roughness of the implants, can be altered or modified in order to achieve the desired effects. These effects include removal torque values, bone-implant contact, biocompatibility and tissue reaction.

## Funding:

None
